# Hemodynamic Performance of Sutureless vs. Conventional Bioprostheses for Aortic Valve Replacement: The 1-Year Core-Lab Results of the Randomized PERSIST-AVR Trial

**DOI:** 10.3389/fcvm.2022.844876

**Published:** 2022-02-18

**Authors:** Theodor Fischlein, Elena Caporali, Federico M. Asch, Ferdinand Vogt, Francesco Pollari, Thierry Folliguet, Utz Kappert, Bart Meuris, Malakh L. Shrestha, Eric E. Roselli, Nikolaos Bonaros, Olivier Fabre, Pierre Corbi, Giovanni Troise, Martin Andreas, Frederic Pinaud, Steffen Pfeiffer, Sami Kueri, Erwin Tan, Pierre Voisine, Evaldas Girdauskas, Filip Rega, Julio García-Puente, Laurent De Kerchove, Roberto Lorusso

**Affiliations:** ^1^Cardiac Surgery, Klinikum Nürnberg, Paracelsus Medical University, Nuremberg, Germany; ^2^Department of Cardiology, Istituto Cardiocentro Ticino, Lugano, Switzerland; ^3^Department of Cardiac Surgery, Istituto Cardiocentro Ticino, Lugano, Switzerland; ^4^Department of Cardio-Thoracic Surgery, Heart and Vascular Centre, Maastricht University Medical Centre (MUMC+), Maastricht, Netherlands; ^5^MedStar Health Research Institute, Washington Hospital Center, Washington D.C., DC, United States; ^6^Cardiac Surgery Unit, Hôpital Henri Mondor, Université Paris 12, Créteil, France; ^7^Herzzentrum Dresden GmbH Universitätsklinik, Dresden, Germany; ^8^Cardiac Surgery Unit, UZ Gasthuisberg Leuven, Leuven, Belgium; ^9^Cardiothoracic and Vascular Surgery, Hannover Medical School, Hannover, Germany; ^10^Heart, Vascular and Thoracic Institute, Cleveland Clinic, Cleveland, OH, United States; ^11^Department of Cardiac Surgery, Medical University of Innsbruck, Innsbruck, Austria; ^12^Lens Hospital and Bois Bernard Private Hospital, Lens, France; ^13^Poitiers University Hospital, Poitiers, France; ^14^Fondazione Poliambulanza Istituto Ospedaliero, Brescia, Italy; ^15^Department of Cardiac Surgery, Medical University of Vienna, Vienna, Austria; ^16^Department of Cardiac Surgery, University Hospital Angers, Angers, France; ^17^University Heart Center Freiburg, Bad Krozingen, Germany; ^18^Catharina Ziekenhuis, Eindhoven, Netherlands; ^19^Division of Cardiac Surgery, Quebec Heart and Lung Institute, Quebec, QC, Canada; ^20^University Heart Center Hamburg, Universitätsklinikum Hamburg Eppendorf (UKE), Hamburg, Germany; ^21^University General Hospital Virgen de la Arrixaca, Murcia, Spain; ^22^Cliniques Universitaires Saint-Luc (UCL), Bruxelles, Belgium; ^23^Cardiac Surgery, Cardiovascular Research Institute Maastricht (CARIM), Maastricht, Netherlands

**Keywords:** aortic stenosis, aortic valve replacement, sutureless aortic valves, stented bioprostheses, randomized trial

## Abstract

**Objective:**

Sutureless aortic valves are an effective option for aortic valve replacement (AVR) showing non-inferiority to standard stented aortic valves for major cardiovascular and cerebral events at 1-year. We report the 1-year hemodynamic performance of the sutureless prostheses compared with standard aortic valves, assessed by a dedicated echocardiographic core lab.

**Methods:**

Perceval Sutureless Implant vs. Standard Aortic Valve Replacement (PERSIST-AVR) is a prospective, randomized, adaptive, open-label trial. Patients undergoing AVR, as an isolated or combined procedure, were randomized to receive a sutureless [sutureless aortic valve replacement (Su-AVR)] (*n* = 407) or a stented sutured [surgical AVR (SAVR)] (*n* = 412) bioprostheses. Site-reported echocardiographic examinations were collected at 1 year. In addition, a subgroup of the trial population (Su-AVR *n* = 71, SAVR = 82) had a complete echocardiographic examination independently assessed by a Core Lab (MedStar Health Research Institute, Washington D.C., USA) for the evaluation of the hemodynamic performance.

**Results:**

The site-reported hemodynamic data of stented valves and sutureless valves are stable and comparable during follow-up, showing stable reduction of mean and peak pressure gradients through one-year follow-up (mean: 12.1 ± 6.2 vs. 11.5 ± 4.6 mmHg; peak: 21.3 ± 11.4 vs. 22.0 ± 8.9 mmHg). These results at 1-year are confirmed in the subgroup by the core-lab assessed echocardiogram with an average mean and peak gradient of 12.8 ± 5.7 and 21.5 ± 9.1 mmHg for Su-AVR, and 13.4 ± 7.7 and 23.0 ± 13.0 mmHg for SAVR. The valve effective orifice area was 1.3 ± 0.4 and 1.4 ± 0.4 cm^2^ at 1-year for Su-AVR and SAVR. These improvements are observed across all valve sizes. At 1-year evaluation, 91.3% (*n* = 42) of patients in Su-AVR and 82.3% in SAVR (*n* = 51) groups were free from paravalvular leak (PVL). The rate of mild PVL was 4.3% (*n* = 2) in Su-AVR and 12.9% (*n* = 8) in the SAVR group. A similar trend is observed for central leak occurrence in both core-lab assessed echo groups.

**Conclusion:**

At 1-year of follow-up of a PERSIST-AVR patient sub-group, the study showed comparable hemodynamic performance in the sutureless and the stented-valve groups, confirmed by independent echo core lab. Perceval sutureless prosthesis provides optimal sealing at the annulus with equivalent PVL and central regurgitation extent rates compared to sutured valves. Sutureless valves are therefore a reliable and essential technology within the modern therapeutic possibilities to treat aortic valve disease.

## Introduction

Aortic valve stenosis is the most common valvular heart disease in adults. The early recognition and management of this pathology are very important because untreated asymptomatic and symptomatic severe diseases result in poor outcomes if managed with conservative treatment. In these patients, therefore, aortic valve replacement (AVR) represents the treatment of choice (1–4). New prostheses have been developed to minimize the surgical risk in patients with multiple comorbidities and to reduce operating times. Sutureless prostheses have shown promising results in terms of mortality, morbidity, and hemodynamic performance (5–7). Several studies have demonstrated that sutureless valves decrease the cardiopulmonary bypass (CPB) and aortic cross-clamping (ACC) times, facilitating minimally and conventional invasive cardiac surgery (5–8), but no randomized controlled trials have directly compared this technology with traditional stented valves (9). Recently, the Perceval Sutureless Implant vs. Standard AVR (PERSIST-AVR) trial has been published with overall 1-year results demonstrating no substantial difference between sutureless vs. conventional tissue valves for isolated or combined surgical AVR (SAVR) (10, 11). The present study addressed the hemodynamic performance at 1 year in the sutureless and the stented-valve groups, in a limited patient subgroup analyzed by the enrollment sites and by an independent echo core lab.

## Methods

Details about the design of the PERSIST-AVR trial have been previously published (10). The PERSIST-AVR trial is a multicenter, prospective, randomized, open-label, noninferiority trial with an adaptive design, conceived to demonstrate the noninferiority of the Perceval sutureless prosthesis compared with standard stented aortic bioprostheses in patients with severe symptomatic aortic valve stenosis. The study was approved by the local Ethical Committees and Institutional Review Boards (IRB: local no. METC151138, national no. NL56524.068), and the participants gave written informed consent before enrolment in the study.

From March 2016 to September 2018, adult patients with severe symptomatic aortic valve stenosis who were candidates for isolated or combined SAVR procedure for native aortic valve disease were prospectively enrolled at 47 international centers and randomized (1:1 blocked randomization) with the Perceval sutureless valve (Perceval, Corcym S.r.l., Saluggia, Italy) or a standard stented aortic bioprosthesis (selected on the basis of surgeon's discretion).

Clinical and echocardiographic follow-up was performed at hospital discharge, between 1 and 3 months, and at 1 year. Site-reported echocardiographic examinations were collected and, in addition, a subgroup of the trial population had a complete echocardiographic examination independently assessed by a Core Lab (MedStar Health Research Institute, Washington D.C., USA) for the evaluation of the hemodynamic performance. Peak and mean aortic gradients were obtained by using Continuous Wave Doppler (CW) using the simplified Bernoulli equation.

### Statistical Analysis

Analyses were conducted on the per-protocol population (excluding patients with Major Deviation) (10, 11) with Core-lab assessed data.

Descriptive statistics have been calculated, using as reference the number of subjects in the relevant analysis population according to the nature of each parameter as follows: categorical variables are reported as absolute and relative frequencies; for quantitative (continuous) parameters, a number of subjects with available and missing data, mean, standard deviation (SD), median, quartiles (Q1-Q3), and extreme values [Minimum; Maximum] are reported. The aortic mean and peak pressure gradients (P2) were also analyzed considering the left ventricular outflow tract (LVOT) gradients (P1) and calculated using the modified Bernoulli equation (P2-P1).

## Results

A total of 914 patients were enrolled, and 910 underwent randomization at 47 international centers. The population in the primary outcome analysis (per protocol) involved 819 patients, 407 in the sutureless group and 412 in the stented group (11).

The subgroup of the trial per-protocol population with complete echocardiographic examination independently assessed by a Core Lab comprises 71 patients implanted with Perceval sutureless prosthesis and 82 implanted with a stented valve; patients were selected according to the site capability to follow the echo core lab protocol by the study.

Preoperative patient profiles and operative characteristics demonstrated no differences in pre-operative risk (EUROSCORE II/STS SCORE) and baseline characteristics between patients implanted with the Perceval sutureless prosthesis and the stented valve cohorts (Tables 1, 2). A mini-sternotomy approach was used in 49.3% of the Perceval group and 51.2% of the stented group; the number of concomitant procedures was also well balanced between the two cohorts (Table 3).

**Table 1:** Patients' baseline characteristics.

	**PERCEVAL (*n =* 71)**	**STENTED (*n =* 82)**	***p*-value**
Age (*y*)	74.7 ± 5.7	74.7.0 ± 4.5	>0.05
Female sex	32 (45.1%)	35 (42.7%)	>0.05
Hypertension	53 (74.6%)	68 (82.9%)	>0.05
Dyslipidemia	40 (56.3%)	57 (69.5%)	>0.05
Diabetes	20 (28.2%)	23 (28.0%)	>0.05
Tobacco user	12 (16.9%)	28 (34.1%)	<0.05
Chronic lung disease	5 (7.0%)	5 (6.1%)	>0.05
Pulmonary hypertension	1 (1.4%)	2 (2.4%)	>0.05
Neoplasia	7 (9.9%)	4 (4.9%)	>0.05
Peripheral vascular disease	6 (8.5%)	9 (11.0%)	>0.05
Carotid artery disease	3 (4.2%)	9 (11.0%)	>0.05
Angina	7 (9.9%)	6 (7.3%)	>0.05
Coronary artery disease	28 (39.4%)	29 (35.4%)	>0.05
Previous PCI	5 (7.0%)	11 (13.4%)	>0.05
Myocardial infarction	3 (4.2%)	3 (3.7%)	>0.05
Heart failure	1 (1.4%)	2 (2.4%)	>0.05
Transient ischemic attack	1 (1.4%)	0 (0.0%)	>0.05
Stroke	4 (5.6%)	3 (3.7%)	>0.05
Previous CABG	1 (1.4%)	1 (1.2%)	>0.05
Pre-existing pacemaker	1 (1.4%)	1 (1.2%)	>0.05
STS score	2.5 ± 2.2	2.1 ± 1.3	>0.05
EuroSCORE II	1.9 ± 1.1	1.8 ± 1.1	>0.05

*Values are mean ± standard deviation, n (%)*.

*PCI, percutaneous coronary intervention. CABG, coronary artery bypass graft. STS, society of thoracic surgeons*.

**Table 2:** Preoperative echocardiographic features.

	**PERCEVAL (*n =* 71)**	**STENTED (*n =* 82)**	***p*-value**
Mean pressure gradient (mmHg)	52.1 ± 15.2	46.6 ± 11.3	0.0146
Peak pressure gradient (mmHg)	82.7 ± 24.9	75.8 ± 17.5	0.0575
Effective orifice area (cm^2^)	0.7 ± 0.2	0.7 ± 0.2	1.0000
Left Ventricular Ejection Fraction (%)	59.9 ± 10.7	60.7 ± 9.6	>0.05

*Values are mean ± standard deviation, n (%)*.

**Table 3:** Intraoperative characteristics.

	**PERCEVAL (*n =* 71)**	STENTED ^*^(*n =* 82)	***p*-value**
Full sternotomy	36 (50.7%)	40 (48.8%)	1.000
Ministernotomy	35 (49.3%)	42 (51.2%)	1.000
Bicuspid aortic valve^†^	12 (16.9%)	9 (11.0%)	0.4084
Concomitant CABG	20 (28.2%)	24 (29.3%)	1.000
Concomitant Septal myectomy	6 (8.5%)	4 (4.9%)	0.5117
Concomitant Aortic annulus enlargement	0 (0.0%)	1 (1.2%)	1.000
Concomitant AF treatment and PFO closure	5 (7.0%)	6 (7.3%)	1.000
Valve size			
S (21 mm)	7 (9.9%)	NA	
M (23 mm)	21 (29.6%)	NA	
L (25 mm)	30 (42.3%)	NA	
XL (27 mm)	13 (18.3%)	NA	
19 mm	NA	1 (1.2%)	
21 mm	NA	24 (29.3%)	
23 mm	NA	37 (45.1%)	
25 mm	NA	17 (20.7%)	
27 mm	NA	3 (3.7%)	
Total mean valve size	24.4 ± 1.8	22.9 ± 1.7	

*Values are mean ± standard deviation, n (%)*.

† *Sievers type 1 only allowed per protocol. NA, not applicable. CABG, coronary artery bypass graft. AF, atrial fibrillation. PFO, patent foramen ovale*.

* *Stented valve model described in Supplementary Material*.

The site-reported hemodynamic data of the stented valves and sutureless valves were comparable at one-year follow-up (Table 4).

**Table 4:** Hemodynamic data up to 1-year visit (site-reported).

	**1 year**		***p*-value**
	**PERCEVAL**	**STENTED**	
Mean Gradient (mmHg)	11.5 ± 4.6	12.1 ± 6.2	0.6092
Peak Gradient (mmHg)	22.0 ± 8.9	21.3 ± 11.4	0.6961
EOA (cm^2^) (mean±SD)	1.6 ± 0.5	1.8 ± 0.6	0.0569
EOAi (cm^2^/m^2^) (mean±SD)	0.9 ± 0.2	0.9 ± 0.3	1.0000
Left ventricular ejection fraction (mean±SD)	62.1 ± 8.1	61.1 ± 8.4	0.4964
Left ventricular mass (g) (mean±SD)	198.8 ± 75.9	202.1 ± 74.8	0.8223
Paravalvular leak	*N =* 63	*N =* 74	1.000
None/Trace	61 (96.8)	71 (95.9)	
Mild	2 (3.2)	2 (2.7)	
Moderate/Severe	0 (0.0)	1 (1.4)	
Central leak	*N =* 63	*N =* 74	0.7101
None/Trace	62 (98.4)	73 (98.6)	
Mild	1 (1.6)	0 (0.0)	
Moderate/Severe	0 (0.0)	1 (1.4)	

*Values are mean ± standard deviation, n (%). EOA, effective orifice area index. EOAi, effective orifice area index indexed to body surface area*.

These results at 1 year are confirmed in the subgroup of core-lab assessed echo (Table 5), with an average mean and peak gradient at 1 year of 12.8 ± 5.7 and 21.5 ± 9.1 mmHg for sutureless AVR (Su-AVR), and 13.4 ± 7.7 and 23.0 ± 13.0 mmHg for SAVR. Valve effective orifice area was 1.3 ± 0.4 and 1.4 ± 0.4 cm^2^ at 1 year for Su-AVR and SAVR. These improvements are observed across all valve sizes (more details in the Supplementary Material). At 1-year evaluation, 91.3% (*n* = 42) of the patients in the Su-AVR group and 82.3% in the SAVR group (*n* = 51) were free from paravalvular leak (PVL), while 87.0% (*n* = 40) of the patients in the Su-AVR group and 82.3% in the SAVR group (*n* = 51) were free from a central leak. The rate of mild PVL was 4.3% (*n* = 2) in the Su-AVR and 12.9% (*n* = 8) in the SAVR group, respectively.

**Table 5:** Hemodynamic data up to 1-year visit (core-lab assessed).

	**1 year**		***p*-value**
	**PERCEVAL**	**STENTED**	
Mean Gradient [mmHg] (P2)	12.8 ± 5.7	13.4 ± 7.7	0.6445
Peak Gradient [mmHg] 4(VAO)2	21.5 ± 9.1	23.0 ± 13.0	0.4854
Mean Gradient [mmHg] (P2 -P1)	10.0 ± 5.3	11.1 ± 6.8	0.3881
Peak Gradient [mmHg] 4(V2A- V2L)	16.7 ± 8.2	19.2 ± 11.8	0.2371
EOA (cm^2^) (mean±SD)	1.3 ± 0.4	1.4 ± 0.4	0.3069
EOAi (cm^2^/m^2^) (mean±SD)	0.7 ± 0.2	0.7 ± 0.2	1.0000
Left ventricular ejection fraction (mean±SD)	63.0 ± 5.5	64.0 ± 5.6	0.5567
Left ventricular mass (g) (mean±SD)	163.8 ± 45.5	175.2 ± 45.1	0.3883
Left ventricular mass index (g/m^2^) (mean±SD)	91.0 ± 17.6	91.2 ± 21.4	0.9711
Paravalvular leak	*N =* 46	*N =* 62	0.3148
None/Trace	42 (91.3)	51 (82.3)	
Mild	2 (4.3)	8 (12.9)	
Moderate/Severe	0 (0.0)	0 (0.0)	
Not evaluable	2 (4.3)	3 (4.8)	
Central leak	*N =* 46	*N =* 62	0.8419
None/Trace	40 (87.0)	51 (82.3)	
Mild	4 (8.7)	8 (12.9)	
Moderate/Severe	0 (0.0)	0 (0.0)	
Not evaluable	2 (4.3)	3 (4.8)	

*Values are mean ± standard deviation, n (%). EOA, effective orifice area. EOAi, effective orifice area index indexed to body surface area. Peak and mean aortic gradients were obtained by using Continuous Wave Doppler (CW) using the simplified Bernoulli equation*.

## Discussion

This is a sub-analysis of the first randomized, controlled study (PERSIST-AVR trial) comparing sutureless vs. conventional stented bioprostheses for isolated SAVR (10, 11). On-site and core-lab echocardiographic findings, in a limited patient subgroup, were assessed and compared regarding hemodynamic performances and prosthesis-related regurgitation. The primary endpoint of this study demonstrates that the hemodynamic data of stented valves and sutureless valves were comparable up to 1-year follow-up, showing a stable reduction of mean and peak pressure gradients through 1-year follow-up. Furthermore, as a secondary endpoint, no difference in terms of para-valvular or central valve regurgitation was found between groups.

The use of sutureless bioprostheses has been widely reported to reduce procedure times in aortic valve replacement, both with conventional or minimally invasive approaches (6–8). Despite the extensive experience with Su-AVR (5–8, 12), no randomized, prospective, controlled studies have been performed comparing conventional vs. sutureless-based AVR. Based on this lack, the PERSIST-AVR trial, consisting of a prospective, randomized controlled study in patients undergoing isolated AVR or combined procedure with sutureless or stented bioprostheses, was conducted in 47 centers worldwide (10). The one-year results have shown non-inferiority between Su-AVR and SAVR regarding Major Adverse Cardiac and Cerebrovascular Events (MACCE) at 1-year follow-up (11). However, besides general and main clinical outcomes, several other aspects have been addressed by the PERSIST-AVR trial, including valve hemodynamic assessment at discharge and during the follow-up, with programmed postoperative consultation (10). These investigations included valve-related hemodynamic assessment, also addressing the incidence and extent of implanted bioprosthesis-related regurgitation, already highlighted by the Registry-based analysis (9). Indeed, as well-known, trans-catheter valves, due to the lack of suture-based implantation, may experience the presence of various ranges of valvular regurgitation, mostly para-valvular, but also centrally located. This complication has affected mainly percutaneous trans-catheter valve implantation but has been observed also in the surgical approach with rapid deployment and sutureless prosthesis (9). The reasons for such post-implant regurgitation account for the presence of uneven surface that can lead to PVL or for an incomplete annular sealing due to incorrect valve sizing and also partially retained and unresected calcification (in Su-AVR procedures) (13, 14). As a matter of fact, such a hemodynamic dysfunction has never been shown to be of substantial importance after Su-AVR, but a large Registry analysis has recently shown not-reassuring data about this phenomenon in sutureless valves. The present analysis demonstrated that sutureless valve implantation is associated with a similar rate and extent of either paravalvular or central regurgitation compared to stented bioprostheses. A recent study (6) demonstrated satisfactory hemodynamic performance with the Perceval sutureless bioprosthesis with a stable reduction in gradients in all valve sizes and a stable increase in the valve-effective orifice area, with significant regression in the left ventricular (LV) mass throughout the 5 years of follow-up. Moreover, a low incidence of PVL was reported, showing that the sutureless valve ensures correct sealing at the level of the aortic annulus. Similar results have also been shown (8) in a huge cohort of patients treated with Perceval sutureless prosthesis implanted through a right anterior mini-thoracotomy approach. Mean pressure gradients and LV mass, as well as the diameters, decreased significantly from preoperative values to follow-up. Moderate PVL occurred in one patient only from the sub-group, without hemolysis or symptoms, and therefore not requiring treatments.

Same excellent hemodynamic performances are coming from the largest single-center cohort of patients with the Perceval sutureless bioprosthesis. The mean pressure gradient, the LV ejection fraction, and mass decreased significantly from the preoperative value to follow-up (*p* < 0.001). Moderate paravalvular leakage occurred in only 3 patients without hemolysis who did not require any treatment; while the other 2 patients with paravalvular leak were reoperated for incomplete expansion of the bioprosthesis (12).

Regarding the overall hemodynamic performance, the PERSIST-AVR findings, at 1-year from the surgical procedure, have shown an effective reduction of transvalvular aortic gradients, with a progressive improvement from hospital discharge to 1-year follow-up, and no significant difference between the sutureless and the sutured groups.

The use of core-lab investigation may play a critical role in such investigations, providing an independent assessment from individual site analysis. The analysis of a subgroup of PERSIST-AVR patients with such an independent assessment showed, notably, no difference, also in terms of prosthesis-related leakage, either paravalvular or central.

Continued investigations are warranted to further and more thoroughly explain these outcomes in the sutureless valve cohort.

### Limits of the Study

The findings of this controlled, randomized study were obtained through a multicenter, worldwide study representing a wide clinical experience. The choice of the stented valve was at the discretion of the operating physician, and the surgical techniques or influence of specific tissue valves employed was not considered in this sub-analysis, for which a limited number of patients were enrolled. Furthermore, the original study was not powered for this *ad hoc* analysis, and a limited number of patients were included in the core-lab assessment.

## Conclusion

This study showed comparable 1-year hemodynamic performances in the sutureless and stented-valve groups, confirmed by an independent echo core lab. Perceval sutureless bioprosthesis provides optimal sealing at the annulus site with equivalent PVL rates of sutured valves. Sutureless valves are a reliable and essential technology in modern therapeutic options for the treatment of aortic valve disease. No difference was found with regards to central or paravalvular regurgitation, although a favorable trend was shown in the sutureless valve, indicating a correct sealing at the aortic annulus.

## Data Availability Statement

The raw data supporting the conclusions of this article will be made available by the authors, without undue reservation.

## Ethics Statement

The study was reviewed and approved by the local Ethical Committees and Institutional Review Boards (IRB: Local No. METC151138, National No. NL56524.068) and the participants gave written informed consent before enrolment to participate in the study.

## Author Contributions

TFi, EC, and RL designed the research and have been accountable for its supervision. TFi, EC, FA, FV, FPo, and TFo analyzed and interpreted the data and wrote the paper. UK, BM, MS, and ER provided assistance on data curation. NB, OF, PC, and GT provided intellectual inputs to the clinical study. MA, FPi, and SP have been accountable for the methodology. SK, ET, PV, and EG generated clinical statistics data. FR, JG-P, and LD provided technical assistance. All authors contributed to the article and approved the submitted version.

## Funding

The PERSIST-AVR Trial is funded by Corcym S.r.l.

## Conflict of Interest

UK was employed by Herzzentrum Dresden GmbH Universitätsklinik. This study received funding from Corcym S.r.l. The funder had the following involvement with the study: all trial-related activities and participated in site selection, data monitoring, trial management, and statistical analysis. TFi: consultant CORCYM and BioStable. TFo: consultant CORCYM (Steering Committee). BM and MS: consultant CORCYM Steering Committee and Proctor. ER: consultant CORCYM (Steering Committee and Proctor), speaker for Abbott, consultant, speaker and investigator for Edwards and Medtronic. NB: educational grants: Edwards Lifesciences and CORCYM, Speaker Honoraria: Edwards Lifesciences, CORCYM and Medtronic. OF, GT, SP, SK, JG-P: consultant CORCYM (Proctor). MA: consultant Abbott and Edwards (Proctor), advisor Medtronic. FR: consultant CORCYM and AtriCure (Proctor), Research Support Recipient Medtronic. RL: Consultant Medtronic, LivaNova, CORCYM and Getinge (honoraria paid to the Maastricht University) and Member of the Medical Advisory Board for Eurosets (honoraria paid to the Maastricht University). FA has no personal conflict of interest but directs an academic Core laboratory carrying institutional contracts (MedStar Health) for his work with Corcym/Livanova, Edwards, Medtronic, Boston Scientific, Abbott, Foldax, Biotronik. The remaining authors declare that the research was conducted in the absence of any commercial or financial relationships that could be construed as a potential conflict of interest.

## Publisher's Note

All claims expressed in this article are solely those of the authors and do not necessarily represent those of their affiliated organizations, or those of the publisher, the editors and the reviewers. Any product that may be evaluated in this article, or claim that may be made by its manufacturer, is not guaranteed or endorsed by the publisher.

## Abbreviations

ACC: aortic cross-clamping
CPB: cardiopulmonary bypass
PERSIST-AVR: PERceval Sutureless Implant vs. Standard Aortic Valve Replacement
SAVR: surgical aortic valve replacement
Su-AVR: sutureless aortic valve replacement
EOA: effective orifice area
EOAi: effective orifice area index.
