# Supplementary material for: Hemodynamic Performance of Sutureless vs. Conventional Bioprostheses for Aortic Valve Replacement: The 1-Year Core-Lab Results of the Randomized PERSIST-AVR Trial
Source: Front Cardiovasc Med. 2022 Feb 18;9:844876. doi: 10.3389/fcvm.2022.844876 (PMC8894864; doi:10.3389/fcvm.2022.844876)

Supplementary Material

Echocardiographic Protocol - Core-Lab
Operative Manual

CO-Principal Investigator
Neil Weissman, MD
Cardiovascular Core Laboratories
Washington Hospital Center/Medstar Health Research Institute
100 Irving Street, NW
Suite EB 5123
Washington, DC 20010
Phone: 202 877 0223
Fax: 202 877 0206
E-mail: neil.j.weissman@medstar.net

CO-Principal Investigator
Federico Asch, MD
Cardiovascular Core Laboratories
Washington Hospital Center/Medstar Health Research Institute
100 Irving Street, NW
Suite EB 5123
Washington, DC 20010
Phone: 202 877 0223
Fax: 202 877 0206
E-mail: neil.j.weissman@medstar.net

Echocardiography Core-Lab Manager
Therese Tupas-Habib, RDCS
Cardiovascular Core Laboratories
Washington Hospital Center/Medstar Health Research Institute
100 Irving Street, NW
Suite EB 5123
Washington, DC 20010
Phone: 202 877 8130
Fax: 202 877 0206
E-mail: maria.t.tupas-habib@medstar.net

1 BACKGROUND

Hemodynamic performance of prosthetic heart valves is usually assessed by flow velocities, pressure gradients, effective orifice area and degree of regurgitation. The purpose of this 2D echo-Doppler study is to demonstrate the effectiveness of the valve based on its hemodynamic performance. The procedures and echo parameter calculation methods presented in this protocol provide a standardized method to collect echo data that will be comparable among investigative centres and can be pooled for analysis and interpretation as recommended by regulatory guidance documents.

This protocol is similar to prior studies for consistency in data acquisition and management. This protocol outlines the standard transducer positions, viewing planes and parameters collected using 2D/M-Mode, pulsed wave (PW), continuous wave (CW) and color flow Doppler for prostheses implanted in the aortic position using the transthoracic approach.

This document should be used as an operative manual to obtain high quality echocardiographic studies and transmit the recorded examinations to the Echo Core Laboratory.

It is of primary importance that the echo studies should be technically adequate for quantitative analysis. For this purpose, the following are recommended:

1. appropriate training of the echocardiographers/sonographers;
2. adequate echo machine (contemporary machine acquired within the last 7 years);
3. digital acquisition and storage of the echo studies using DICOM format (or Philips proprietary file format) to preserve image quality and assure readability of the files.

2 ECHOCARDIOGRAPHIC EXAMINATION

2.1 General Principles

The echocardiographic examinations should be performed paying attention to patient's needs. It may be unnecessary to underline the fact that patient care and comfort come first.

In addition, patient cooperation is a prerequisite to obtaining high quality transthoracic echocardiographic studies. To reduce intra-patient variability, all examinations should be performed by the same echocardiographer/sonographer and the same echo machine.

2.2 Patient Preparation

For the TTE examination, the patient should be placed in the left lateral position. Before starting the echo examination, the patient's blood pressure, weight and height should be obtained and documented.

2.3 Echocardiographic Instrumentation

After positioning electrodes on the patient's chest, it will be necessary to optimize the electrocardiographic signal to obtain a QRS complex of adequate size that is clearly visualized on the monitor screen. Recordings of Doppler and M-mode tracings should be performed at 50 or 100 mm/s speed to be able to acquire 2-3 cardiac cycles in each still frame.

For transthoracic studies, the use of 2.5 MHz or higher transducers is required. We recommend the use of a transducer with the highest possible frequency to obtain the best image resolution and an adequate resolution to obtain a good definition of endocardial borders.

Tissue harmonic modality is required to optimize the image, especially for assessment of left ventricular volumes.

2.4 Echocardiographic Examination

To ensure that the highest quality echocardiographic images are acquired and stored, digital capture and storage of the echo studies using DICOM format (or Philips proprietary file format) is required. Only one CD-ROM for each echo study of a single patient should be used.
Each echo examination should contain the **Patient's Study ID number** and the **date of examination**, as they are reported on the Echo CRF. **To comply with HIPPA and other privacy regulations, do not use patient name.**
For color Doppler images, optimize the color sector to the minimum size that shows the whole jet area and maintains adequate frame rate. To ensure intra-patient reproducibility of color flow recordings, the same color map should be maintained in serial studies of the same patient. Color gain should be the highest, providing that no motion artefact is produced by cardiac anatomic structures and there is no speckling superimposed on the image. Wall filters should be set at the lowest level at which cardiac structure motion artefacts are eliminated. Zero velocity line should be placed in the mid of the colour spectrum and the Nyquist limit should be above 50 cm/s.

For each view, cine-loop should be recorded acquiring **3 cardiac cycles in patients with sinus rhythm and 5 cardiac cycles in patients with atrial fibrillation**. This can be accomplished by setting the loop record to 3 beats or multiple single or double beats of the same view can be recorded. For M-mode and Doppler tracings, still frames containing at least 3 cardiac cycles are required.

2.4.1 Transthoracic echocardiographic examination - views

The required echocardiographic recordings for transthoracic studies are listed below:

Parasternal Long Axis (PLAX) View
- 2D image of left ventricle and aortic root
- Zoom image of LVOT
- High parasternal of the aortic root
- Color flow Doppler of mitral and aortic valves

Right Ventricle Inflow (RVIF) View
- 2D image of RV inflow
- Color Doppler of Tricuspid Valve
- Obtain continuous wave (CW) Doppler of tricuspid regurgitation

Parasternal Short Axis (PSAX) View
- 2D image at basal level
- Zoom 2D image of aortic valve
- Color Doppler of aortic and tricuspid valves
- Color Doppler and continuous wave (CW) Doppler of tricuspid regurgitation
- Short axis of LV at papillary muscle level and apex; LV should be circular not elliptical

Parasternal Short Axis (PSAX) View - Aortic Valve Post Implant

- Zoom 2D image of aortic valve
- Color Doppler to assess regurgitation location and severity

Apical 4 chamber (AP4) view

- 2D image of all 4 chambers
- Optimize image of left ventricle to visualize endocardial border delineation; LV must be centered and not foreshortened
- 2D image of entire left atrium
- Color Doppler of mitral and tricuspid valves; Nyquist settings above 50 cm/s.
- CW of TR jet if applicable
- Pulsed wave (PW) Doppler of mitral inflow with sample volume placed at the tip of the mitral leaflet
- Pulsed tissue Doppler of medial and lateral mitral annulus velocity; record at least 3 cardiac cycles

Apical 5 (AP5) chamber view

- 2D apical 5 chambers
- Color Doppler of aortic and mitral valves
- PW Doppler of LVOT; sample volume should be placed 3-5 mm proximal to aortic valve
- CW Doppler through the aortic valve

Apical 2 (AP2) chamber view

- 2D image of apical 2 chambers
- Optimize image of left ventricle; endocardial border should be visualized; image must be centered and not foreshortened
- 2D image of entire left atrium
- Color Doppler of mitral valve

Apical 3 (AP3) chamber view

- 2D image of apical 3 chambers view
- 2D image of aortic valve
- Color Doppler of aortic and mitral valves
- PW Doppler of LVOT
- CW Doppler through aortic valve

Subcostal View

- 2D image of Subcostal 4 chambers view
- Color Doppler of interatrial septum
- 2D image of inferior vena cava in long axis
- M-mode of IVC; place M-mode cursor 1.0-2.0 cm from the junction with the right atrium; subject should take a brief sniff; record M-mode recordings of at least 3 cardiac cycles

Non-Imaging Transducer Recordings

The following views should be attempted with a non-imaging (Pedoff) transducer. The screen should be annotated indicating which view is being attempted.

- **Suprasternal Notch ("SSN")**

- Place transducer in the suprasternal notch
- Record at least 3 beats of transaortic flow

- **Right Parasternal Window ("RPara")**
  - Place the transducer in the 3rd-5th right intercostal space
  - Record at least 3 beats of transaortic flow

- **Subcostal Window ("Sub")**
  - Place the transducer below the xiphoid process
  - Record at least 3 beats of transaortic flow

Native and prosthetic valve function will be assessed calculating effective orifice area, peak and mean gradients, degree of regurgitation, cardiac index, cardiac output and performance index (for valve prosthesis only).
Therefore, for the parasternal long-axis view the operator must zoom in on the LVOT to clearly delineate the LVOT and aortic valve and from the apical views, the left ventricular outflow and transaortic flow Doppler tracings should be optimized for aortic valve area calculation.
The hemodynamic impact of valve disease can be reflected by pulmonary artery pressure. Systolic pulmonary artery pressure will be estimated using CW Doppler interrogation of tricuspid regurgitation jet to calculate right atrio-ventricular gradient. Therefore, care should be taken to record the highest possible tricuspid regurgitant jet velocity.

3 ECHO EXAM AND CASE REPORT FORM

The echo exam performed should be recorded using DICOM format (or Philips proprietary file format) and a labeled to identify the patient ID, exam date and study interval.
After completion of the echocardiographic assessment, please complete the Echo Case Report Form (CRF) which is a checklist and documentation that the required views were performed. If one of the views was not performed, an explanation should be provided.
The Echo CRF must be signed and dated by the echocardiographer/sonographer who performed the echocardiographic exam.
The Echo examination and Echo CRFs will be sent directly to the Core lab upon completion. A copy of the Echo CRF must accompany the echo exam to the Core lab.

4 Supplementary Tables and Figures

4.1 Supplementary Tables

Supplementary Table 1. Hemodynamic data: Sutureless preoperative to 1-year visit overall and by valve size (site reported data. Per-Protocol population with also Core-Lab assessment)

	Pre-operative		Discharge		1−3 months		1 year	
	**Patients (N)**	**Mean Gradient [mmHg]**	**Patients (N)**	**Mean Gradient [mmHg]**	**Patients (N)**	**Mean Gradient [mmHg]**	**Patients (N)**	**Mean Gradient [mmHg]**
Size S/21	7		7		7		6	
Mean±SD		58.9±26.4		13.0±3.8		11.9±3.5		13.9±6.2
Median [Q1; Q3]		50.0 [44.0; 65.6]		13.0 [9.0; 16.5]		13.0 [8.1; 15.0]		14.0 [11.0; 18.0]
Size M/23	21		20		20		19	
Mean±SD		53.4±18.9		15.4±5.6		12.7±4.4		12.6±5.4
Median [Q1; Q3]		52.0 [38.0; 71.0]		16.9 [11.0; 18.0]		12.5 [9.7; 16.0]		12.0 [9.0; 15.0]
Size L/25	29		27		29		25	
Mean±SD		49.5±8.9		13.4±4.6		11.0±4.6		11.0±4.0
Median [Q1; Q3]		49.0 [43.0; 54.0]		14.0 [11.0; 16.0]		10.0 [8.0; 12.1]		11.0 [7.0; 14.0]
Size XL/27	13		12		11		10	
Mean±SD		51.8±12.4		9.9±2.6		10.4±4.1		9.0±2.2
Median [Q1; Q3]		50.0 [44.0; 56.0]		10.0 [8.0; 12.0]		10.0 [7.0; 13.0]		8.5 [8.0; 10.0]
All								
Mean±SD		52.1±15.2		13.3±4.8		11.5±4.4		11.5±4.6
Median [Q1; Q3]		50.0 [43.0; 59.0]		13.0 [10.0; 16.7]		11.0 [8.1; 14.0]		10.6 [8.0; 14.0]
	**Patients (N)**	**Peak Gradient [mmHg]**	**Patients (N)**	**Peak Gradient [mmHg]**	**Patients (N)**	**Peak Gradient [mmHg]**	**Patients (N)**	**Peak Gradient [mmHg]**
Size S/21	7		7		7		5	
Mean±SD		93.4±48.0		21.9±6.2		20.5±7.5		28.9±7.1
Median [Q1; Q3]		74.0 [68.0; 91.8]		22.0 [18.0; 27.2]		21.0 [12.1; 25.3]		30.0 [22.2; 31.0]
Size M/23	21		19		20		20	
Mean±SD		83.2±29.1		27.4±10.6		23.0±6.2		24.3±10.4
Median [Q1; Q3]		79.0 [58.0; 100.0]		26.0 [19.0; 37.0]		23.0 [19.0; 26.3]		23.5 [18.0; 29.4]
Size L/25	29		27		29		26	
Mean±SD		80.8±14.5		24.0±7.7		19.9±7.2		20.5±8.0
Median [Q1; Q3]		75.0 [71.4; 90.0]		25.0 [20.0; 29.0]		19.0 [15.0; 22.0]		20.0 [14.0; 25.0]
Size XL/27	11		11		9		8	
Mean±SD		79.7±20.2		18.7±5.8		20.4±7.4		16.7±4.8
Median [Q1; Q3]		77.0 [64.0; 89.0]		17.0 [16.0; 24.0]		17.0 [16.0; 22.0]		17.8 [14.0; 19.0]
All								
Mean±SD		82.7±24.9		23.9±8.6		21.0±6.9		22.0±8.9
Median [Q1; Q3]		76.5 [69.0; 93.0]		24.0 [17.9; 29.0]		20.1 [16.0; 25.0]		20.0 [16.0; 28.0]
	**Patients (N)**	**EOA (cm^2)**	**Patients (N)**	**EOA (cm^2)**	**Patients (N)**	**EOA (cm^2)**	**Patients (N)**	**EOA (cm^2)**
Size S/21	5		3		5		4	
Mean±SD		06±0.3		1.5±0.3		1.3±0.5		1.4±0.6
Median [Q1; Q3]		0.6 [0.3; 0.7]		1.4 [1.2; 1.8]		1.4 [0.9; 1.6]		1.3 [0.9; 1.9]
Size M/23	18		15		16		17	
Mean±SD		0.6±0.2		1.7±0.8		1.7±0.5		1.5±0.4
Median [Q1; Q3]		0.6 [0.5; 0.8]		1.5 [1.1; 2.3]		1.5 [1.4; 1.9]		1.4 [1.2; 1.7]
Size L/25	28		22		26		22	
Mean±SD		0.7±0.2		1.9±0.6		1.7±0.5		1.7±0.5
Median [Q1; Q3]		0.7 [0.5; 0.9]		1.7 [1.6; 2.2]		1.6 [1.4; 2.0]		1.6 [1.3; 2.0]
Size XL/27	12		6		7		7	
Mean±SD		0.6±0.2		2.2±0.5		1.9±0.5		1.7±0.4
Median [Q1; Q3]		0.7 [0.5; 0.9]		2.1 [1.8; 2.8]		1.8 [1.4; 2.5]		1.6 [1.5; 2.2]
All								
Mean±SD		0.7±0.2		1.8±0.7		1.7±0.5		1.6±0.5
Median [Q1; Q3]		0.7 [0.5; 0.8]		1.7 [1.4; 2.2]		1.6 [1.4; 1.9]		1.6 [1.3; 1.9]
	**Patients (N)**	**EOAi (cm^2/m^2)**	**Patients (N)**	**EOAi (cm^2/m^2)**	**Patients (N)**	**EOAi (cm^2/m^2)**	**Patients (N)**	**EOAi (cm^2/m^2)**
Size S/21	4		3		5		4	
Mean±SD		0.3±0.2		0.8±0.1		0.8±0.2		0.8±0.3
Median [Q1; Q3]		0.3 [0.2; 0.4]		0.8 [0.7; 0.9]		0.8 [0.6; 1.0]		0.8 [0.6; 1.1]
Size M/23	17		15		15		17	
Mean±SD		0.4±0.1		1.0±0.5		0.9±0.2		0.9±0.2
Median [Q1; Q3]		0.3 [0.3; 0.4]		0.9 [0.7; 1.4]		0.9 [0.7; 1.1]		0.8 [0.7; 1.0]

	Pre-operative		Discharge		1−3 months		1 year	
Size L/25<br>Mean±SD<br>Median [Q1; Q3]	22	0.3±0.1<br>0.4 [0.3; 0.4]	18	1.0±0.4<br>1.0 [0.8; 1.2]	22	0.9±0.2<br>0.9 [0.7; 1.1]	20	0.9±0.3<br>0.9 [0.7; 1.0]
Size XL/27<br>Mean±SD<br>Median [Q1; Q3]	9	0.3±0.1<br>0.4 [0.2; 0.5]	6	1.2±0.2<br>1.2 [1.0; 1.3]	7	1.0±0.4<br>1.0 [0.6; 1.5]	6	1.0±0.2<br>0.8 [0.8; 1.2]
All<br>Mean±SD<br>Median [Q1; Q3]		0.3±0.1<br>0.3 [0.3; 0.4]		1.0±0.4<br>1.0 [0.8; 1.2]		0.9±0.2<br>0.9 [0.7; 1.1]		0.9±0.2<br>0.9 [0.7; 1.0]
	**Patients (N)**	**Left ventricular ejection (%)**	**Patients (N)**	**Left ventricular ejection (%)**	**Patients (N)**	**Left ventricular ejection (%)**	**Patients (N)**	**Left ventricular ejection (%)**
Size S/21<br>Mean±SD<br>Median [Q1; Q3]	7	65.0±4.2<br>65.0 [61.3; 70.0]	7	58.7±4.1<br>60.0 [58.0; 60.0]	7	62.4±10.3<br>62.0 [60.0; 65.0]	7	64.4±4.2<br>65.0 [60.0; 66.0]
Size M/23<br>Mean±SD<br>Median [Q1; Q3]	21	62.5±8.4<br>62.0 [56.0; 70.0]	21	60.2±10.2<br>60.0 [55.0; 65.0]	20	62.9±8.2<br>61.5 [60.0; 69.5]	20	60.2±7.5<br>60.5 [57.5; 64.5]
Size L/25<br>Mean±SD<br>Median [Q1; Q3]	25	58.2±7.7<br>60.0 [55.0; 64.0]	25	54.8±10.0<br>55.0 [51.5; 60.0]	24	58.8±9.1<br>60.5 [54.5; 64.0]	23	63.5±9.7<br>62.0 [57.0; 72.0]
Size XL/27<br>Mean±SD<br>Median [Q1; Q3]	12	60.4±15.2<br>62.5 [52.5; 69.5]	11	58.1±6.5<br>58.0 [55.0; 60.0]	11	60.6±7.9<br>59.0 [57.0; 60.0]	9	60.8±7.2<br>60.0 [57.0; 65.0]
All<br>Mean±SD<br>Median [Q1; Q3]		60.7±9.6<br>60.0 [55.0; 68.0]		57.6±9.2<br>60.0 [53.0; 60.0]		60.9±8.7<br>60.0 [57.0; 65.0]		62.1±8.1<br>61.0 [57.0; 66.0]
	**Patients (N)**	**Left ventricular mass (g)**	**Patients (N)**	**Left ventricular mass (g)**	**Patients (N)**	**Left ventricular mass (g)**	**Patients (N)**	**Left ventricular mass (g)**
Size S/21<br>Mean±SD<br>Median [Q1; Q3]	4	145.3±22.4<br>144.0 [128.5; 162.0]	3	139.3±16.6<br>137.0 [124.0; 157.0]	2	128.0±14.1<br>128.0 [118.0; 138.0]	4	99.3±20.4<br>94.0 [84.0; 114.5]
Size M/23<br>Mean±SD<br>Median [Q1; Q3]	18	190.4±60.8<br>170.8 [150.0; 244.0]	13	198.4±105.2<br>170.0 [132.1; 212.0]	16	202.9±50.9<br>192.0 [163.5; 252.0]	14	188.4±57.0<br>168.5 [145.0; 225.0]
Size L/25<br>Mean±SD<br>Median [Q1; Q3]	28	251.2±90.7<br>226.5 [186.5; 322.5]	24	252.1±103.7<br>218.0 [176.2; 323.0]	24	230.9±86.3<br>222.0 [166.5; 298.5]	21	220.7±90.9<br>187.0 [161.0; 267.0]
Size XL/27<br>Mean±SD<br>Median [Q1; Q3]	10	210.0±73.7<br>208.4 [152.0; 248.0]	7	195.2±55.9<br>169.0 [149.7; 233.0]	8	220.1±46.3<br>215.0 [193.5; 259.5]	9	208.1±35.0<br>220.8 [189.0; 230.0]
All<br>Mean±SD<br>Median [Q1; Q3]		219.0±82.5<br>202.0 [160.0; 254.5]		221.6±99.4<br>186.0 [149.7; 248.0]		216.1±71.1<br>208.5 [166.0; 257.0]		198.8±75.9<br>183.5 [145.5; 228.0]

Supplementary Table 2. Hemodynamic data: Stented preoperative to 1-year visit overall and by valve size (site reported data. Per-Protocol population with also Core-Lab assessment)

	Pre-operative		Discharge		1−3 months		1 year	
	**Patients (N)**	**Mean Gradient [mmHg]**	**Patients (N)**	**Mean Gradient [mmHg]**	**Patients (N)**	**Mean Gradient [mmHg]**	**Patients (N)**	**Mean Gradient [mmHg]**
19 mm	1		1		1		1	
Mean±SD		68.0		27.5		23.0		27.3
Median [Q1; Q3]		NA		NA		NA		NA
21 mm	23		21		20		20	
Mean±SD		49.5±11.6		13.5±6.1		13.6±6.9		15.5±7.3
Median [Q1; Q3]		48.0 [41.0; 61.0]		14.0 [8.0; 17.0]		11.7 [8.9; 15.2]		14.5 [10.5; 17.5]
23 mm	35		37		34		32	
Mean±SD		44.4±9.8		11.7±5.0		10.3±5.1		11.4±4.7
Median [Q1; Q3]		44.0 [40.0; 48.0]		11.0 [8.0; 14.0]		10.0 [5.0; 14.3]		12.0 [8.0; 14.0]
25 mm	16		14		12		13	
Mean±SD		46.8±13.0		9.8±4.2		6.5±2.7		8.1±3.6
Median [Q1; Q3]		46.0 [37.5; 54.0]		8.0 [7.0; 11.0]		5.5 [5.0; 8.5]		8.0 [6.0; 11.0]
27 mm	3		3		2		2	
Mean±SD		43.0±11.0		12.0±8.0		7.5±3.5		7.5±3.5
Median [Q1; Q3]		43.0 [32.0; 54.0]		12.0 [4.0; 20.0]		7.5 [5.0; 10.0]		7.0 [5.0; 10.0]
All								
Mean±SD		46.6±11.3		12.1±5.6		10.7±6.0		12.1±6.2
Median [Q1; Q3]		44.0 [40.0; 51.0]		11.0 [8.0; 16.0]		10.0 [6.0; 14.3]		11.0 [8.0; 15.0]
	**Patients (N)**	**Peak Gradient [mmHg]**	**Patients (N)**	**Peak Gradient [mmHg]**	**Patients (N)**	**Peak Gradient [mmHg]**	**Patients (N)**	**Peak Gradient [mmHg]**
19 mm	1		1		1		1	
Mean±SD		112.0		42.4		34.0		45.4
Median [Q1; Q3]		NA		NA		NA		NA
21 mm	24		20		20		19	
Mean±SD		79.9±19.1		24.0±11.4		24.4±12.7		28.2±14.0
Median [Q1; Q3]		79.0 [64.1; 95.5]		20.5 [15.0; 31.3]		20.5 [16.0; 27.0]		25.0 [20.0; 32.0]
23 mm	35		34		34		32	
Mean±SD		73.1±15.3		21.1±8.6		18.1±8.3		20.5±8.7
Median [Q1; Q3]		70.0 [66.0; 77.0]		20.5 [15.0; 25.0]		19.5 [10.0; 24.0]		20.0 [12.5; 26.2]
25 mm	17		17		15		16	
Mean±SD		75.1±17.2		15.5±6.6		12.6±6.2		14.0±6.3
Median [Q1; Q3]		72.0 [66.0; 89.0]		14.0 [12.0; 16.0]		10.0 [8.0; 18.0]		13.0 [10.5; 17.5]
27 mm	3		2		3		2	
Mean±SD		67.7±22.0		17.0±14.1		12.7±6.0		14.0±7.1
Median [Q1; Q3]		57.0 [53.0; 93.0]		17.0 [7.0; 27.0]		12.0 [7.0; 19.0]		14.0 [9.0; 19.0]
All								
Mean±SD		75.8±17.5		20.8±9.8		18.7±10.2		21.3±11.4
Median [Q1; Q3]		72.5 [65.6; 86.0]		18.0 [14.0; 26.0]		18.0 [10.0; 24.0]		19.5 [12.0; 27.0]
	**Patients (N)**	**EOA (cm^2)**	**Patients (N)**	**EOA (cm^2)**	**Patients (N)**	**EOA (cm^2)**	**Patients (N)**	**EOA (cm^2)**
19 mm	1		1		1		NA	
Mean±SD		0.5		1.1		1.2		NA
Median [Q1; Q3]		NA		NA		NA		
21 mm	21		13		14		15	
Mean±SD		0.6±0.2		1.4±0.3		1.5±0.4		1.3±0.3
Median [Q1; Q3]		0.7 [0.5; 0.8]		1.5 [1.4; 1.5]		1.4 [1.1; 2.0]		1.3 [1.1; 1.5]
23 mm	33		25		29		31	
Mean±SD		0.7±0.2		1.9±0.8		1.8±0.7		1.8±0.5
Median [Q1; Q3]		0.7 [0.5; 0.9]		1.7 [1.6; 2.2]		1.6 [1.4; 2.1]		1.7 [1.4; 2.2]
25 mm	17		13		12		12	
Mean±SD		0.7±0.2		2.2±0.4		2.3±0.6		2.0±0.6
Median [Q1; Q3]		0.8 [0.6; 0.8]		2.2 [2.0; 2.3]		2.1 [1.8; 2.8]		2.1 [1.5; 2.5]
27 mm	3		1		2		2	
Mean±SD		0.7±0.1		2.3		2.5±0.7		2.8±0.9
Median [Q1; Q3]		0.7 [0.6; 0.8]		NA		2.5 [2.0; 3.0]		2.8 [2.1; 3.4]
All								
Mean±SD		0.7±0.2		1.8±0.6		1.9±0.7		1.8±0.6
Median [Q1; Q3]		0.7 [0.6; 0.8]		1.7 [1.5; 2.2]		1.8 [1.4; 2.1]		1.7 [1.3; 2.2]
	**Patients (N)**	**EOAi (cm^2/m^2)**	**Patients (N)**	**EOAi (cm^2/m^2)**	**Patients (N)**	**EOAi (cm^2/m^2)**	**Patients (N)**	**EOAi (cm^2/m^2)**
19 mm	1		NA		NA		NA	
Mean±SD		0.3		NA		NA		NA
Median [Q1; Q3]		NA						
21 mm	16		11		12		15	
Mean±SD		0.3±0.1		0.8±0.2		0.9±0.3		0.7±0.2
Median [Q1; Q3]		0.4 [0.3; 0.4]		0.8 [0.8; 0.9]		0.8 [0.7; 1.1]		0.7 [0.6; 0.7]
23 mm	31		24		29		31	
Mean±SD		0.4±0.1		1.0±0.3		1.1±0.5		1.0±0.3
Median [Q1; Q3]		0.4 [0.3; 0.5]		0.9 [0.8; 1.1]		0.9 [0.7; 1.2]		0.9 [0.8; 1.3]
25 mm	13		12		12		12	
Mean±SD		0.4±0.1		1.1±0.2		1.2±0.3		1.1±0.3

	Pre-operative		Discharge		1−3 months		1 year	
Median [Q1; Q3]		0.4 [0.3; 0.4]		1.0 [1.0; 1.1]		1.1 [1.0; 1.4]		1.1 [0.9; 1.3]
27 mm<br>Mean±SD<br>Median [Q1; Q3]	2	0.3±0.0<br>0.3 [0.3; 0.3]	1	1.3<br>NA	2	1.2±0.5<br>1.2 [0.8; 1.5]	2	1.3±0.6<br>1.3 [0.9; 1.7]
All<br>Mean±SD<br>Median [Q1; Q3]		0.4±0.1<br>0.4 [0.3; 0.4]		1.0±0.3<br>0.9 [0.8; 1.1]		1.0±0.5<br>1.0 [0.7; 1.2]		0.9±0.3<br>0.9 [0.7; 1.2]
	**Patients (N)**	**Left ventricular ejection (%)**	**Patients (N)**	**Left ventricular ejection (%)**	**Patients (N)**	**Left ventricular ejection (%)**	**Patients (N)**	**Left ventricular ejection (%)**
19 mm<br>Mean±SD<br>Median [Q1; Q3]	1	60.0<br>NA	1	65.0<br>NA	1	56.0<br>NA	1	65.0<br>NA
21 mm<br>Mean±SD<br>Median [Q1; Q3]	24	61.6±14.7<br>64.5 [55.5; 68.9]	20	58.3±12.5<br>60.0 [50.0; 64.3]	19	59.8±4.3<br>60.0 [58.0; 62.0]	20	61.4±6.7<br>60.0 [59.9; 65.0]
23 mm<br>Mean±SD<br>Median [Q1; Q3]	35	60.6±6.7<br>60.0 [59.0; 64.0]	36	58.6±7.1<br>60.0 [54.5; 61.0]	34	58.3±7.0<br>60.0 [55.0; 62.0]	32	59.8±9.1<br>60.0 [54.5; 66.0]
25 mm<br>Mean±SD<br>Median [Q1; Q3]	14	55.3±11.8<br>58.5 [43.0; 60.0]	13	56.7±7.2<br>58.0 [53.0; 60.0]	13	63.8±10.7<br>65.0 [60.0; 68.0]	13	63.9±9.8<br>60.0 [59.0; 70.0]
27 mm<br>Mean±SD<br>Median [Q1; Q3]	2	60.0<br>NA	2	55.0<br>NA	3	63.0±5.2<br>60.0 [60.0; 69.0]	2	60.0<br>NA
All<br>Mean±SD<br>Median [Q1; Q3]		59.9±10.7<br>60.0 [56.0; 65.0]		58.2±8.8<br>60.0 [54.0; 61.0]		59.9±7.3<br>60.0 [58.0; 62.5]		61.1±8.4<br>60.0 [59.0; 66.0]
	**Patients (N)**	**Left ventricular mass (g)**	**Patients (N)**	**Left ventricular mass (g)**	**Patients (N)**	**Left ventricular mass (g)**	**Patients (N)**	**Left ventricular mass (g)**
19 mm<br>Mean±SD<br>Median [Q1; Q3]	1	121.7<br>NA	NA	NA	1	114.0<br>NA	1	124.6<br>NA
21 mm<br>Mean±SD<br>Median [Q1; Q3]	19	233.1±108.3<br>234.0 [166.0; 255.0]	15	170.0±47.3<br>167.0 [148.0; 200.0]	13	165.0±49.9<br>155.0 [144.6; 170.0]	13	189.3±53.8<br>184.4 [157.0; 222.0]
23 mm<br>Mean±SD<br>Median [Q1; Q3]	28	216.5±94.2<br>201.5 [149.7; 269.8]	21	229.5±90.3<br>192.0 [174.0; 244.0]	32	196.3±55.0<br>203.5 [151.2; 228.0]	27	192.6±73.6<br>177.0 [139.0; 219.0]
25 mm<br>Mean±SD<br>Median [Q1; Q3]	16	240.4±96.6<br>223.0 [181.3; 316.5]	14	239.9±109.8<br>228.0 [153.0; 287.0]	13	250.6±80.9<br>237.0 [186.0; 304.0]	16	230.3±89.8<br>211.5 [172.0; 281.0]
27 mm<br>Mean±SD<br>Median [Q1; Q3]	3	231.3±58.2<br>226.0 [176.0; 292.0]	2	236.5±40.3<br>236.5 [208.0; 265.0]	2	205.5±29.0<br>205.5 [185.0; 226.0]	2	228.5±54.4<br>228.5 [190.0; 267.0]
All<br>Mean±SD<br>Median [Q1; Q3]		226.2±96.5<br>212.0 [165.0; 271.5]		215.4±88.2<br>190.5 [165.5; 246.0]		200.2±65.8<br>200.0 [148.0; 229.0]		202.1±74.8<br>189.0 [148.0; 228.0]

Supplementary Table 3. Hemodynamic data: Sutureless preoperative to 1-year visit overall and by valve size (Core lab data. Per-Protocol population)

	Discharge		1–3 months		1 year	
	**Patients (N)**	**Mean Gradient [mmHg] (P2)**	**Patients (N)**	**Mean Gradient [mmHg] (P2)**	**Patients (N)**	**Mean Gradient [mmHg] (P2)**
Size S/21<br>Mean±SD<br>Median [Q1; Q3]	6	13.1±3.7<br>12.8 [11.8; 14.5]	6	13.0±3.2<br>13.2 [11.2; 14.1]	4	13.8±4.3<br>13.6 [10.6; 16.9]
Size M/23<br>Mean±SD<br>Median [Q1; Q3]	20	15.5±5.6<br>14.6 [11.0; 20.0]	20	13.3±3.8<br>13.8 [9.9; 16.2]	18	14.7±7.4<br>11.7 [8.9; 20.0]
Size L/25<br>Mean±SD<br>Median [Q1; Q3]	27	14.5±4.0<br>14.3 [12.0; 17.0]	26	11.3±3.7<br>10.6 [9.3; 13.3]	19	11.4±4.2<br>10.9 [8.2; 14.8]
Size XL/27<br>Mean±SD<br>Median [Q1; Q3]	8	10.0±2.9<br>10.5 [7.5; 12.4]	6	12.1±4.3<br>11.3.0 [8.9; 12.3]	7	11.6±4.4<br>10.7 [9.4; 14.8]
All<br>Mean±SD<br>Median [Q1; Q3]		14.1±4.7<br>13.5 [11.0; 17.0]		12.3±3.8<br>11.6 [9.4; 14.5]		12.8±5.7<br>11.7 [8.9; 15.9]
	**Patients (N)**	**Peak Gradient [mmHg] 4(VAO)2**	**Patients (N)**	**Peak Gradient [mmHg] 4(VAO)2**	**Patients (N)**	**Peak Gradient [mmHg] 4(VAO)2**
Size S/21<br>Mean±SD<br>Median [Q1; Q3]	6	21.3±6.1<br>21.2 [18.7; 25.7]	6	21.5±5.5<br>23.1 [18.3; 24.6]	4	23.5±7.7<br>24.1 [17.4; 29.6]
Size M/23<br>Mean±SD<br>Median [Q1; Q3]	20	25.6±9.2<br>24.1 [18.4; 34.0]	20	22.4±5.8<br>23.5 [16.8; 27.8]	18	24.1±11.7<br>20.4 [15.7; 31.3]
Size L/25<br>Mean±SD<br>Median [Q1; Q3]	27	23.7±6.2<br>24.8 [18.4; 27.2]	26	18.3±5.5<br>16.9 [14.9; 21.0]	19	19.3±6.9<br>20.5 [14.0; 23.0]
Size XL/27<br>Mean±SD<br>Median [Q1; Q3]	8	16.5±5.1<br>17.6 [12.7; 20.0]	6	20.8±7.2<br>19.9 [16.7; 20.6]	7	19.5±7.0<br>18.9 [14.0; 24.4]
All<br>Mean±SD<br>Median [Q1; Q3]		23.1±7.6<br>21.5 [18.3; 27.2]		20.3±5.9<br>19.4 [16.0; 24.8]		21.5±9.1<br>20.5 [15.0; 27.2]
	**Patients (N)**	**Mean Gradient [mmHg] (P2 -P1)**	**Patients (N)**	**Mean Gradient [mmHg] (P2 -P1)**	**Patients (N)**	**Mean Gradient [mmHg] (P2 -P1)**
Size S/21<br>Mean±SD<br>Median [Q1; Q3]	5	9.8±3.5<br>9.7 [8.9; 10.5]	5	9.5±3.0<br>10.4 [6.6; 10.6]	2	8.1±3.2<br>8.1 [5.8; 10.3]
Size M/23<br>Mean±SD<br>Median [Q1; Q3]	11	13.2±5.1<br>15.0 [7.4; 17.6]	15	10.0±3.2<br>10.0 [6.9; 13.4]	17	11.4±6.8<br>9.1 [7.1; 14.6]
Size L/25<br>Mean±SD<br>Median [Q1; Q3]	17	10.2±3.2<br>10.1 [7.3; 12.3]	25	8.4±3.3<br>8.0 [7.0; 10.0]	14	8.8±3.5<br>8.6 [5.4; 11.8]
Size XL/27<br>Mean±SD<br>Median [Q1; Q3]	7	8.0±2.3<br>8.2 [6.9; 9.6]	6	9.4±3.9<br>9.0 [6.0; 10.8]	7	9.5±4.4<br>8.3 [6.9; 12.4]
All<br>Mean±SD<br>Median [Q1; Q3]		10.6±4.0<br>9.7 [7.4; 13.2]		9.1±3.3<br>8.8 [6.6; 11.0]		10.3±5.3<br>9.1 [6.3; 12.8]
	**Patients (N)**	**Peak Gradient [mmHg] 4(V2A- V2L)**	**Patients (N)**	**Peak Gradient [mmHg] 4(V2A- V2L)**	**Patients (N)**	**Peak Gradient [mmHg] 4(V2A- V2L)**
Size S/21<br>Mean±SD<br>Median [Q1; Q3]	5	15.2±5.8<br>14.8 [14.0; 18.7]	2	15.6±5.5<br>17.5 [10.7; 20.0]	2	13.3±5.4<br>13.3 [9.4; 17.1]
Size M/23<br>Mean±SD<br>Median [Q1; Q3]	11	22.3±8.7<br>22.3 [13.2; 31.9]	6	17.1±5.2<br>17.8 [12.5; 21.3]	17	18.8±10.4<br>14.6 [12.4; 24.5]
Size L/25<br>Mean±SD<br>Median [Q1; Q3]	17	17.2±5.6<br>18.4 [12.7; 21.2]	5	13.8±4.9<br>12.8 [11.4; 15.7]	14	15.2±5.6<br>15.6 [9.9; 19.9]
Size XL/27<br>Mean±SD<br>Median [Q1; Q3]	7	13.3±4.6<br>13.4 [10.5; 17.6]	7	16.7±6.3<br>16.2 [12.0; 18.2]	7	15.9±7.1<br>13.7 [10.1; 20.4]
All<br>Mean±SD<br>Median [Q1; Q3]		17.7±7.0<br>17.4 [12.6; 22.2]		15.3±5.3<br>15.2 [11.5; 20.0]		16.7±8.2<br>14.6 [10.5; 21.3]
	**Patients (N)**	**EOA (cm^2)**	**Patients (N)**	**EOA (cm^2)**	**Patients (N)**	**EOA (cm^2)**

	Discharge		1–3 months		1 year	
Size S/21<br>Mean±SD<br>Median [Q1; Q3]	5	1.6±0.7<br>1.8 [1.1; 1.9]	4	1.3±0.6<br>1.1 [1.0; 1.7]	2	1.5±0.6<br>1.5 [1.1; 1.9]
Size M/23<br>Mean±SD<br>Median [Q1; Q3]	11	1.3±0.3<br>1.2 [1.0; 1.4]	11	1.4±0.3<br>1.4 [1.3; 1.6]	12	1.3±0.3<br>1.2 [1.1; 1.4]
Size L/25<br>Mean±SD<br>Median [Q1; Q3]	14	1.6±0.4<br>1.6 [1.3; 1.9]	20	1.6±0.4<br>1.6 [1.4; 1.9]	11	1.4±0.4<br>1.4 [1.1; 1.8]
Size XL/27<br>Mean±SD<br>Median [Q1; Q3]	5	1.7±0.4<br>1.8 [1.7; 1.9]	5	1.7±0.5<br>1.5 [1.4; 1.7]	4	1.3±0.3<br>1.5 [1.2; 1.5]
All<br>Mean±SD<br>Median [Q1; Q3]		1.5±0.5<br>1.5 [1.2; 1.9]		1.5±0.4<br>1.5 [1.3; 1.8]		1.3±0.4<br>1.3 [1.3; 1.5]
	**Patients (N)**	**EOAi (cm^2/m^2)**	**Patients (N)**	**EOAi (cm^2/m^2)**	**Patients (N)**	**EOAi (cm^2/m^2)**
Size S/21<br>Mean±SD<br>Median [Q1; Q3]	5	0.9±0.3<br>1.1 [0.6; 1.1]	4	0.8±0.2<br>0.7 [0.6; 0.9]	2	0.7±0.3<br>0.7 [0.5; 0.9]
Size M/23<br>Mean±SD<br>Median [Q1; Q3]	11	0.7±0.2<br>0.7 [0.6; 0.8]	11	0.8±0.2<br>0.8 [0.8; 0.9]	12	0.7±0.2<br>0.7 [0.6; 0.8]
Size L/25<br>Mean±SD<br>Median [Q1; Q3]	14	0.8±0.2<br>0.8 [0.7; 1.0]	20	0.9±0.2<br>0.8 [0.8; 1.0]	11	0.8±0.2<br>0.8 [0.6; 1.0]
Size XL/27<br>Mean±SD<br>Median [Q1; Q3]	5	0.9±0.2<br>0.9 [0.8; 0.9]	5	0.9±0.3<br>0.8 [0.7; 1.0]	4	0.6±0.2<br>0.7 [0.5; 0.7]
All<br>Mean±SD<br>Median [Q1; Q3]		0.8±0.2<br>0.8 [0.6; 1.0]		0.8±0.2<br>0.8 [0.7; 1.0]		0.7±0.2<br>0.7 [0.6; 0.8]
	**Patients (N)**	**Left ventricular ejection (%)**	**Patients (N)**	**Left ventricular ejection (%)**	**Patients (N)**	**Left ventricular ejection (%)**
Size S/21<br>Mean±SD<br>Median [Q1; Q3]	3	65.3±4.7<br>67.0 [60.0; 69.0]	4	63.0±5.3<br>64.0 [59.0; 67.0]	2	62.5±7.8<br>62.5 [57.0; 68.0]
Size M/23<br>Mean±SD<br>Median [Q1; Q3]	11	62.8±9.5<br>65.0 [61.0; 68.0]	13	63.1±6.1<br>64.0 [61.0; 68.0]	8	64.5±5.1<br>66.0 [62.5; 68.0]
Size L/25<br>Mean±SD<br>Median [Q1; Q3]	17	59.0±8.8<br>60.0 [54.0; 66.0]	16	58.1±12.9<br>61.5 [55.5; 66.0]	7	62.6±6.1<br>65.0 [60.0; 67.0]
Size XL/27<br>Mean±SD<br>Median [Q1; Q3]	5	57.8±10.7<br>63.0 [59.0; 64.0]	5	64.2±5.5<br>66.0 [60.0; 68.0]	2	59.0±5.7<br>59.0 [55.0; 63.0]
All<br>Mean±SD<br>Median [Q1; Q3]		60.5±9.1<br>63.5 [58.5; 66.5]		61.1±9.6<br>635 [57.0; 67.0]		63.0±5.5<br>65.0 [60.0; 68.0]
	**Patients (N)**	**Left ventricular mass (g)**	**Patients (N)**	**Left ventricular mass (g)**	**Patients (N)**	**Left ventricular mass (g)**
Size S/21<br>Mean±SD<br>Median [Q1; Q3]	3	123.7±24.6<br>126.0 [98.0; 147.0]	2	129.0±4.2<br>129.0 [126.0; 132.0]	2	146.0±45.3<br>146.0 [114.0; 178.0]
Size M/23<br>Mean±SD<br>Median [Q1; Q3]	6	149.5±33.2<br>149.0 [132.0; 178.0]	7	144.1±41.6<br>142.0 [114.0; 158.0]	8	138.8±15.8<br>137.5 [130.0; 150.0]
Size L/25<br>Mean±SD<br>Median [Q1; Q3]	6	184.5±68.5<br>174.5 [141.0; 207.0]	11	201.2±65.3<br>187.0 [151.0; 273.0]	6	172.3±53.0<br>159.0 [141.0; 181.0]
Size XL/27<br>Mean±SD<br>Median [Q1; Q3]	3	226.3±49.2<br>248.0 [170.0; 261.0]	5	199.6±68.1<br>168.0 [159.0; 212.0]	3	225.7±30.1<br>241.0 [191.0; 245.0]
All<br>Mean±SD<br>Median [Q1; Q3]		169.7±56.8<br>161.5 [132.0; 191.0]		179.1±62.1<br>159.0 [133.0; 212.0]		163.8±45.5<br>150.0 [130.0; 181.0]
	**Patients (N)**	**Left ventricular mass index (g/m^2)**	**Patients (N)**	**Left ventricular mass index (g/m^2)**	**Patients (N)**	**Left ventricular mass index (g/m^2)**
Size S/21<br>Mean±SD<br>Median [Q1; Q3]	3	76.4±19.2<br>70.0 [61.3; 98.0]	2	83.3±1.1<br>83.3 [82.5; 84.0]	2	90.4±20.3<br>90.4 [76.0; 104.7]
Size M/23<br>Mean±SD<br>Median [Q1; Q3]	6	85.5±18.3<br>90.3 [69.5; 98.9]	7	80.7±22.7<br>74.7 [67.1; 94.4]	8	80.8±11.4<br>79.1 [71.9; 88.3]
Size L/25	6		11		6	

	Discharge		1–3 months		1 year	
Mean±SD<br>Median [Q1; Q3]		99.3±31.8<br>96.9 [94.0; 115.0]		105.4±28.4<br>110.0 [88.8; 130.0]		97.6±22.2<br>96.4 [88.2; 100.6]
Size XL/27<br>Mean±SD<br>Median [Q1; Q3]	3	110.1±32.8<br>118.6 [73.9; 137.8]	5	100.8±28.9<br>93.5 [93.3; 111.6]	3	105.6±5.5<br>104.8 [100.5; 111.4]
All<br>Mean±SD<br>Median [Q1; Q3]		92.7±26.5<br>95.3 [70.0; 106.1]		95.8±27.0<br>93.5 [74.7; 111.6]		91.0±17.6<br>88.8 [76.0; 101.3]

Supplementary Table 4. Hemodynamic data: Stented preoperative to 1-year visit overall and by valve size (Core-lab data. Per-Protocol population)

	Discharge		1–3 months		1 year	
	**Patients (N)**	**Mean Gradient [mmHg] (P2)**	**Patients (N)**	**Mean Gradient [mmHg] (P2)**	**Patients (N)**	**Mean Gradient [mmHg] (P2)**
19 mm	1		NA		1	
Mean±SD		24.7		NA		38.8
Median [Q1; Q3]		NA				NA
21 mm	17		16		17	
Mean±SD		13.9±4.6		15.3±7.6		17.1±9.3
Median [Q1; Q3]		13.9 [10.8; 16.7]		14.6 [9.1; 19.1]		16.6 [9.6; 21.2]
23 mm	30		33		30	
Mean±SD		12.3±4.7		10.6±4.5		11.9±5.2
Median [Q1; Q3]		11.9 [9.1; 14.7]		10.9 [6.0; 13.5]		10.3 [8.0; 16.6]
25 mm	15		12		10	
Mean±SD		9.7±5.3		7.4±3.4		10.0±4.3
Median [Q1; Q3]		8.5 [5.8; 11.5]		6.1 [5.4; 9.9]		8.6 [6.4; 14.4]
27 mm	3		2		1	
Mean±SD		10.7±4.0		7.4±4.2		8.2
Median [Q1; Q3]		10.8 [6.7; 14.7]		7.4 [4.4; 10.3]		NA
All						
Mean±SD		12.2±5.1		11.1±5.9		13.4±7.7
Median [Q1; Q3]		11.3 [8.6; 15.3]		10.7 [6.0; 14.3]		11.1 [8.1; 17.1]
	**Patients (N)**	**Peak Gradient [mmHg] 4(VAO)2**	**Patients (N)**	**Peak Gradient [mmHg] 4(VAO)2**	**Patients (N)**	**Peak Gradient [mmHg] 4(VAO)2**
19 mm	1		NA		1	
Mean±SD		38.6		NA		61.6
Median [Q1; Q3]		NA				NA
21 mm	17		16		17	
Mean±SD		23.9±7.8		26.0±12.9		29.5±16.9
Median [Q1; Q3]		24.5 [18.8; 27.9]		24.8 [16.6; 29.7]		26.8 [18.3; 36.1]
23 mm	30		33		30	
Mean±SD		20.6±7.3		18.1±7.2		20.2±8.4
Median [Q1; Q3]		21.1 [14.7; 25.3]		19.0 [10.6; 22.3]		18.6 [12.6; 27.4]
25 mm	15		12		10	
Mean±SD		16.4±7.5		12.9±5.8		17.1±6.7
Median [Q1; Q3]		14.8 [10.4; 20.8]		10.8 [9.0; 17.7]		16.3 [10.7; 24.3]
27 mm	3		2		1	
Mean±SD		18.4±7.6		12.5±7.6		15.1
Median [Q1; Q3]		19.5 [10.4; 25.4]		12.5 [7.1; 17.8]		NA
All						
Mean±SD		20.7±8.1		18.9±9.8		23.0±13.0
Median [Q1; Q3]		20.2 [14.8; 26.1]		18.0 [10.6; 24.7]		19.4 [13.3; 29.1]
	**Patients (N)**	**Mean Gradient [mmHg] (P2 -P1)**	**Patients (N)**	**Mean Gradient [mmHg] (P2 -P1)**	**Patients (N)**	**Mean Gradient [mmHg] (P2 -P1)**
19 mm	NA		NA		NA	
Mean±SD		NA		NA		NA
Median [Q1; Q3]						
21 mm	11		14		16	
Mean±SD		10.7±3.7		12.3±7.1		14.5±9.2
Median [Q1; Q3]		10.4 [8.0; 12.6]		11.0 [6.1; 15.8]		12.6 [7.3; 18.7]
23 mm	20		31		26	
Mean±SD		8.9±4.2		7.4±4.0		10.1±5.0
Median [Q1; Q3]		8.9 [6.6; 10.9]		8.0 [3.9; 10.4]		10.0 [5.5; 13.9]
25 mm	11		12		8	
Mean±SD		7.6±5.8		5.0±3.2		8.5±3.6
Median [Q1; Q3]		6.1 [2.9; 14.7]		4.2 [2.4; 7.4]		8.2 [5.3; 12.0]
27 mm	2		2		1	
Mean±SD		5.8±3.0		4.7±3.3		4.7
Median [Q1; Q3]		5.8 [3.7; 7.9]		4.7 [2.3; 7.0]		NA
All						
Mean±SD		8.9±4.5		8.0±5.3		11.1±6.8
Median [Q1; Q3]		8.5 [6.0; 11.4]		7.3 [4.0; 10.6]		10.3 [5.8; 14.0]
	**Patients (N)**	**Peak Gradient [mmHg] 4(V2A- V2L)**	**Patients (N)**	**Peak Gradient [mmHg] 4(V2A- V2L)**	**Patients (N)**	**Peak Gradient [mmHg] 4(V2A- V2L)**
19 mm	NA		NA		NA	
Mean±SD		NA		NA		NA
Median [Q1; Q3]						
21 mm	11		14		16	
Mean±SD		18.8±6.3		21.4±12.3		25.2±16.7
Median [Q1; Q3]		17.8 [13.0; 23.1]		19.6 [11.1; 24.1]		21.3 [13.6; 31.4]
23 mm	20		31		26	
Mean±SD		15.1±6.6		12.9±6.6		17.1±8.2
Median [Q1; Q3]		15.2 [9.8; 19.0]		12.6 [6.9; 17.9]		17.4 [9.3; 22.9]

	Discharge		1–3 months		1 year	
25 mm<br>Mean±SD<br>Median [Q1; Q3]	11	12.7±8.4<br>10.8 [5.2; 22.3]	12	8.8±5.6<br>7.2 [4.9; 13.0]	8	14.9±5.5<br>15.0 [10.4; 20.4]
27 mm<br>Mean±SD<br>Median [Q1; Q3]	2	9.9±7.1<br>9.9 [4.8; 14.9]	2	8.0±6.1<br>8.0 [3.7; 12.3]	1	8.5<br>NA
All<br>Mean±SD<br>Median [Q1; Q3]		15.2±7.2<br>14.4 [10.4; 19.6]		13.9±9.2<br>12.4 [6.9; 18.5]		19.2±11.8<br>17.3 [9.8; 23.3]
	**Patients (N)**	**EOA (cm²)**	**Patients (N)**	**EOA (cm²)**	**Patients (N)**	**EOA (cm²)**
19 mm<br>Mean±SD<br>Median [Q1; Q3]	NA	NA	NA	NA	NA	NA
21 mm<br>Mean±SD<br>Median [Q1; Q3]	9	1.4±0.2<br>1.4 [1.2; 1.5]	12	1.4±0.3<br>1.4 [1.1; 1.6]	15	1.2±0.2<br>1.2 [1.0; 1.3]
23 mm<br>Mean±SD<br>Median [Q1; Q3]	17	1.7±0.4<br>1.6 [1.5; 2.0]	29	1.8±0.5<br>1.8 [1.5; 2.0]	20	1.5±0.4<br>1.4 [1.2; 1.8]
25 mm<br>Mean±SD<br>Median [Q1; Q3]	9	1.8±0.4<br>1.7 [1.6; 2.0]	10	2.1±0.3<br>2.1 [1.8; 2.4]	5	1.7±0.4<br>1.5 [1.4; 1.7]
27 mm<br>Mean±SD<br>Median [Q1; Q3]	1	2.4<br>NA	2	2.7±0.7<br>2.7 [2.3; 3.2]	1	2.6<br>NA
All<br>Mean±SD<br>Median [Q1; Q3]		1.7±0.4<br>1.6 [14; 2.0]		1.8±0.5<br>1.8 [1.5; 2.1]		1.4±0.4<br>1.3 [1.1; 1.5]
	**Patients (N)**	**EOAi (cm²/m²)**	**Patients (N)**	**EOAi (cm²/m²)**	**Patients (N)**	**EOAi (cm²/m²)**
19 mm<br>Mean±SD<br>Median [Q1; Q3]	NA	NA	NA	NA	NA	NA
21 mm<br>Mean±SD<br>Median [Q1; Q3]	9	0.7±0.1<br>0.7 [0.7; 0.8]	12	0.7±0.2<br>0.7 [0.6; 0.8]	15	0.6±0.1<br>0.6 [0.5; 0.8]
23 mm<br>Mean±SD<br>Median [Q1; Q3]	17	0.9±0.3<br>0.9 [0.8; 1.1]	29	0.9±0.3<br>0.9 [0.7; 1.1]	20	0.8±0.2<br>0.7 [0.6; 0.9]
25 mm<br>Mean±SD<br>Median [Q1; Q3]	9	0.9±0.3<br>0.8 [0.8; 1.0]	10	1.1±0.2<br>1.0 [0.9; 1.2]	5	0.8±0.2<br>0.7 [0.7; 0.8]
27 mm<br>Mean±SD<br>Median [Q1; Q3]	1	1.3<br>NA	2	1.3±0.5<br>1.3 [0.9; 1.7]	1	1.0<br>NA
All<br>Mean±SD<br>Median [Q1; Q3]		0.9±0.3<br>0.8 [0.7; 1.0]		0.9±0.3<br>0.9 [0.7; 1.1]		0.7±0.2<br>0.7 [0.6; 0.8]
	**Patients (N)**	**Left ventricular ejection (%)**	**Patients (N)**	**Left ventricular ejection (%)**	**Patients (N)**	**Left ventricular ejection (%)**
19 mm<br>Mean±SD<br>Median [Q1; Q3]	1	66.0<br>NA	NA	NA<br>NA	1	68.0<br>NA
21 mm<br>Mean±SD<br>Median [Q1; Q3]	9	61.2±7.0<br>63.0 [57.0; 67.0]	11	60.7±6.9<br>63.0 [58.0; 66.0]	6	64.8±5.1<br>66.5 [62.0; 68.0]
23 mm<br>Mean±SD<br>Median [Q1; Q3]	17	59.5±5.3<br>60.0 [59.0; 62.0]	20	60.8±6.3<br>63.0 [56.0; 65.0]	14	62.7±6.2<br>65.0 [60.0; 67.0]
25 mm<br>Mean±SD<br>Median [Q1; Q3]	6	54.0±11.8<br>57.0 [42.0; 62.0]	8	59.9±11.3<br>66.0 [55.0; 67.0]	3	65.3±4.6<br>68.0 [60.0; 68.0]
27 mm<br>Mean±SD<br>Median [Q1; Q3]	1	60.0<br>NA	1	68.0<br>NA	1	70.0<br>NA
All<br>Mean±SD<br>Median [Q1; Q3]		59.2±7.3<br>60.5 [57.0; 65.0]		60.8±7.5<br>63.0 [57.5; 66.0]		64.0±5.6<br>66.0 [62.0; 68.0]
	**Patients (N)**	**Left ventricular mass (g)**	**Patients (N)**	**Left ventricular mass (g)**	**Patients (N)**	**Left ventricular mass (g)**
19 mm<br>Mean±SD<br>Median [Q1; Q3]	1	124.0<br>NA	NA	NA	1	207.0<br>NA

	Discharge		1–3 months		1 year	
21 mm<br>Mean±SD<br>Median [Q1; Q3]	9	152.6±55.2<br>123.0 [115.0; 172.0]	10	166.8±81.8<br>140.0 [106.0; 167.0]	10	159.5±42.7<br>145.5 [134.0; 188.0]
23 mm<br>Mean±SD<br>Median [Q1; Q3]	10	213.4±54.7<br>201.0 [171.0; 246.0]	17	172.3±29.9<br>170.0 [162.0; 193.0]	17	167.4±38.5<br>175.0 [146.0; 191.0]
25 mm<br>Mean±SD<br>Median [Q1; Q3]	6	229.0±79.7<br>210.5 [159.0; 319.0]	5	227.4±60.8<br>198.0 [187.0; 248.0]	5	227.0±42.1<br>241.0 [204.0; 211.0]
27 mm<br>Mean±SD<br>Median [Q1; Q3]	NA	NA	1	356.0<br>NA	NA	NA
All<br>Mean±SD<br>Median [Q1; Q3]		192.5±67.7<br>171.5 [147.0; 246.0]		184.5±64.6<br>170.0 [147.0; 193.0]		175.2±45.1<br>175.0 [140.0; 200.0]
	**Patients (N)**	**Left ventricular mass index (g/m²)**	**Patients (N)**	**Left ventricular mass index (g/m²)**	**Patients (N)**	**Left ventricular mass index (g/m²)**
19 mm<br>Mean±SD<br>Median [Q1; Q3]	1	77.5<br>NA	NA	NA	1	129.4<br>NA
21 mm<br>Mean±SD<br>Median [Q1; Q3]	9	79.6±26.5<br>67.6 [58.5; 90.5]	10	86.6±39.3<br>77.8 [55.8; 91.7]	10	84.5±17.3<br>82.4 [74.4; 94.0]
23 mm<br>Mean±SD<br>Median [Q1; Q3]	10	111.6±30.2<br>103.6 [90.0; 123.0]	17	88.7±17.5<br>91.9 [77.3; 101.6]	17	87.3±20.0<br>92.1 [70.0; 102.2]
25 mm<br>Mean±SD<br>Median [Q1; Q3]	6	121.0±43.0<br>108.1 [84.0; 169.5]	5	114.7±34.1<br>103.9 [99.0; 118.1]	5	110.2±20.1<br>120.5 [102.0; 124.3]
27 mm<br>Mean±SD<br>Median [Q1; Q3]	NA	NA	1	142.4<br>NA	0	228.5±54.4<br>228.5 [190.0; 267.0]
All<br>Mean±SD<br>Median [Q1; Q3]		101.4±35.4<br>92.2 [79.5; 120.5]		93.6±30.1<br>91.7 [77.3; 103.9]		91.2±21.4<br>92.1 [75.0; 106.1]

Supplementary Table 5. Hemodynamic data at discharge and up to 3-months visit (site-reported)

	Discharge		1-3 months	
	**Perceval**	**Stented**	**Perceval**	**Stented**
**Mean Gradient (mmHg)**	13.3±4.8	12.1±5.6	11.5±4.4	10.7±6.0
**Peak Gradient (mmHg)**	23.9±8.6	20.8±9.8	21.0±6.9	18.7±10.2
**EOA (cm^2) (mean±SD)**	1.8±0.7	1.8±0.6	1.7±0.5	1.9±0.7
**EOAi (cm^2/m^2) (mean±SD)**	1.0±0.4	1.0±0.3	0.9±0.2	1.0±0.5
**Left ventricular ejection fraction (mean±SD)**	57.6±9.2	58.2±8.8	60.9±8.7	59.9±7.3
**Left ventricular mass (g) (mean±SD)**	221.6±99.4	215.4±88.2	216.1±71.1	200.2±65.8
**Paravalvular leak**	N=70	N=80	N=69	N=74
**None/Trace**	66 (94.3)	73 (91.3)	65 (94.2)	68 (91.9)
**Mild**	1 (1.4)	0 (0.0)	3 (4.3)	2 (2.7)
**Moderate/Severe**	0 (0.0)	1 (1.3)	0 (0.0)	1 (1.4)
**Not evaluable**	3 (4.3)	6 (7.5)	1 (1.4)	3 (4.0)
**Central leak**	N=70	N=80	N=69	N=74
**None/Trace**	66 (94.3)	74 (92.5)	66 (95.7)	71 (95.9)
**Mild**	1 (1.4)	0 (0.0)	1 (1.4)	0 (0.0)
**Moderate/Severe**	0 (0.0)	0 (0.0)	0 (0.0)	0 (0.0)
**Not evaluable**	3 (4.3)	6 (7.5)	2 (2.9)	3 (4.1)

Values are mean ± standard deviation, n (%). EOA: effective orifice area index. EOAi: effective orifice area index indexed to body surface area.

Supplementary Table 6. Hemodynamic data at discharge and up to 3-months visit (core-lab assessed)

	Discharge		1-3 months	
	**Perceval**	**Stented**	**Perceval**	**Stented**
**Mean Gradient [mmHg] (P2)**	14.1±4.7	12.2±5.1	12.3±3.8	11.1±5.9
**Peak Gradient [mmHg] 4(VAO)2**	23.1±7.6	20.7±8.1	20.3±5.9	18.9±9.8
**Mean Gradient [mmHg] (P2 -P1)**	10.6±4.0	8.9±4.5	9.1±3.3	8.0±5.3
**Peak Gradient [mmHg] 4(V2A- V2L)**	17.7±7.0	15.2±7.2	15.3±5.3	13.9±9.2
**EOA (cm^2) (mean±SD)**	1.5±0.5	1.7±0.4	1.5±0.4	1.8±0.5
**EOAi (cm^2/m^2) (mean±SD)**	0.8±0.2	0.9±0.3	0.8±0.2	0.9±0.3
**Left ventricular ejection fraction (mean±SD)**	60.5±9.1	59.2±7.3	61.1±9.6	60.8±7.5
**Left ventricular mass (g) (mean±SD)**	169.7±56.8	192.5±67.7	179.1±62.1	184.5±64.6
**Left ventricular mass index (g/m^2) (mean±SD)**	92.7±26.5	101.4±35.4	95.8±27.0	93.6±30.1
**Paravalvular leak**	N=65	N=67	N=57	N=62
**None/Trace**	61 (93.9)	57 (86.4)	50 (87.7)	51 (82.2)
**Mild**	1 (1.5)	5 (7.5)	4 (7.0)	8 (12.9)
**Moderate/Severe**	0 (0.0)	0 (0.0)	0 (0.0)	1 (1.6)
**Not evaluable**	3 (4.6)	5 (7.5)	3 (5.3)	2 (3.2)
**Central leak**	N=65	N=67	N=57	N=62
**None/Trace**	61 (93.8)	60 (89.5)	52 (91.2)	58 (93.5)
**Mild**	1 (1.5)	2 (3.0)	2 (3.5)	0 (0.0)
**Moderate/Severe**	0 (0.0)	0 (0.0)	0 (0.0)	2 (3.2)
**Not evaluable**	3 (4.6)	5 (7.5)	3 (5.3)	2 (3.2)

Values are mean ± standard deviation, n (%). EOA: effective orifice area. EOAi: effective orifice area index indexed to body surface area. Peak and mean aortic gradients were obtained by using Continuous Wave Doppler (CW) using the simplified Bernoulli equation.

Supplementary Table 7. Stented valve model

	STENTED (n=82)
**Carpentier-Edwards PERIMOUNT**	3 (3.7)
**Carpentier-Edwards PERIMOUNT Magna**	1 (1.2)
**Carpentier-Edwards PERIMOUNT Magna EASE**	28 (34.1)
**Crown PRT**	25 (30.5)
**Hancock II/Hancock II ULTRA**	6 (7.3)
**Trifecta**	18 (22.0)
**Other**	1 (1.2)

Values are n (%).

4.2 Supplementary Figures

Supplementary Figure 1. Paravalvular leak at discharge, 1-3 months and 1 year follow up

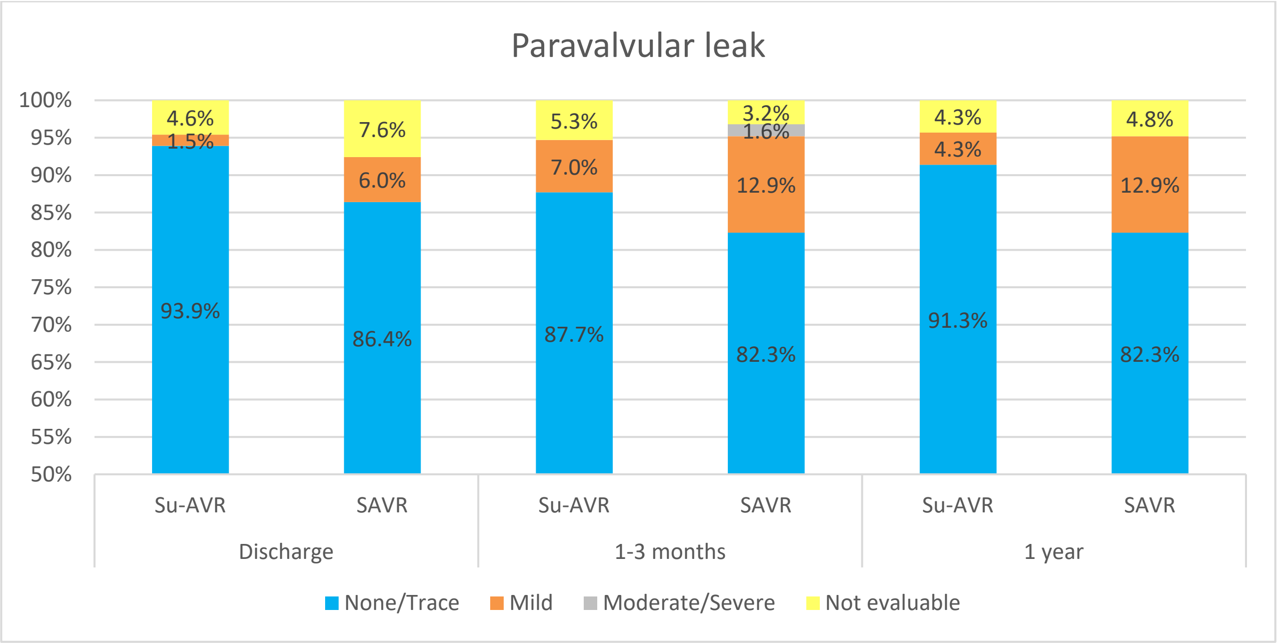

Supplementary Figure 2. Central leak at discharge, 1-3 months and 1 year follow up

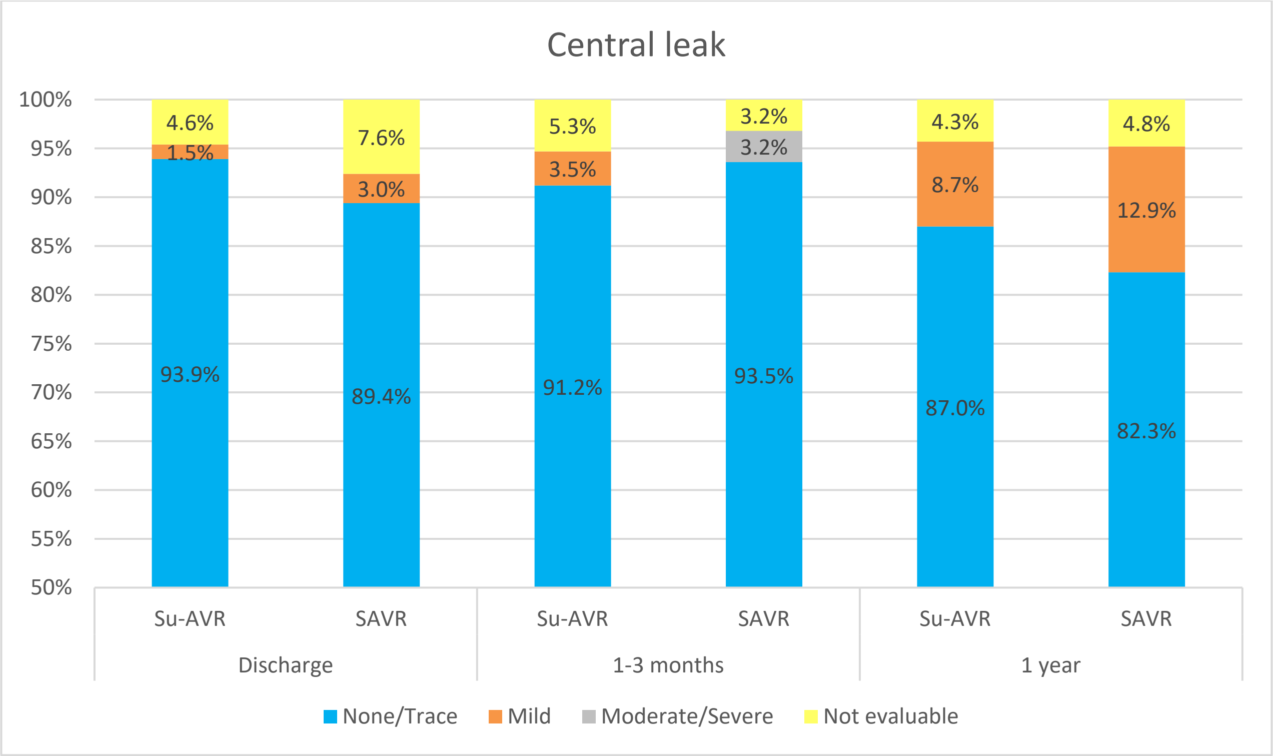

Supplementary Figure 3. Randomization flow chart

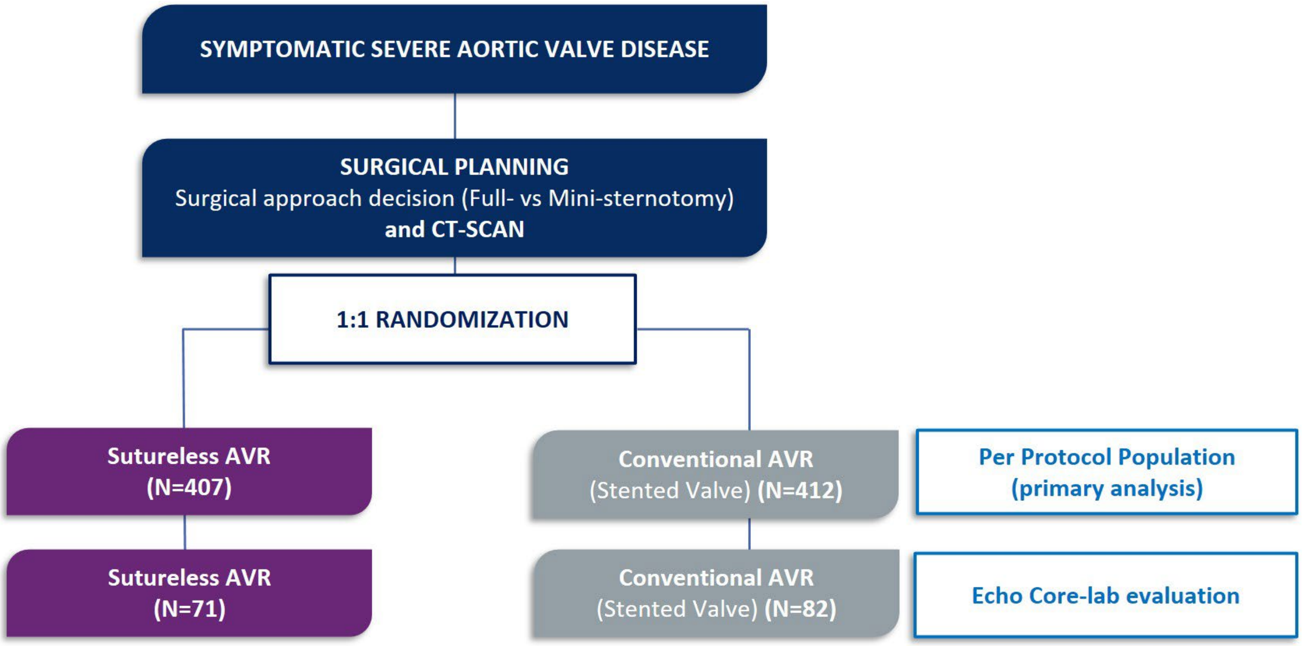

Supplement: Supplementary file 1 [file Data_Sheet_1.pdf]
